# A Comparative Analysis of the Responses of *Lucilia cuprina* (Wiedemann) and *Chrysomya rufifacies* (Macqart) (Calliphoridae) to Different Reflectance Levels of Green and Yellow Light Hues

**DOI:** 10.3390/insects17030283

**Published:** 2026-03-05

**Authors:** Tharindu B. Bambaradeniya, Paola A. Magni, Ian R. Dadour

**Affiliations:** 1 School of Medical, Molecular & Forensic Sciences, Murdoch University, Murdoch, WA 6150, Australia; sritharindu27@gmail.com (T.B.B.);; 2 Harry Butler Institute, Murdoch University, Murdoch, WA 6150, Australia; 3 Source Certain, Wangara, P.O. Box 1570, Wangara, WA 6947, Australia

**Keywords:** Diptera, *Chrysomya rufifacies*, *Lucilia cuprina*, visual cues, reflectance intensity, colour hue

## Abstract

Proximate visual cues play a crucial role for flies (Order: Diptera) in locating suitable foraging and oviposition sites. This study examined the behavioural responses of two sheep myiasis-causing blowfly species in Australia, *Lucilia cuprina* (Wiedemann) and *Chrysomya rufifacies* (Macquart), to six different reflectance levels of green and yellow hues. Both species were influenced primarily by reflectance intensity and proximity to the light source. *Lucilia cuprina* displayed a nonsignificant preference for moderate yellow (*p* = 0.25), whereas *Ch. rufifacies* showed a significant attraction to moderate green (*p* = 0.004) when presented with a two-choice comparison between moderate green and yellow. When exposed to three reflectance levels under each hue, both species responded most strongly to mid-range intensities in green but not yellow, with no significant differences observed among light and dark shades (*p* > 0.05). Zonal analyses revealed a significant aggregation of individuals near the light source (Zone C; *p* < 0.05), indicating that spatial orientation cues may be stronger determinants of attraction. Overall, *L. cuprina* was more responsive to moderate yellow and *Ch. rufifacies* to moderate green, but both species were predominantly guided by light-related spatial and intensity cues. These findings provide valuable insight into the visual ecology of blowflies and may be useful in optimising colour and reflectance parameters in the design of future commercial fly traps.

## Simple Summary

Sheep blow fly strike is a serious problem for sheep welfare worldwide. Two of the most important blow fly species involved are *Lucilia cuprina* (Wiedemann) and *Chrysomya rufifacies* (Macquart). Of the many cues flies use to locate a host, vision is important, with colour being useful to find places for feeding and laying eggs. Commercial fly traps often use bright colours to attract flies, but the most effective colours differ among species and are not well understood, although green and yellow are known to attract flies. The aim of this study was to examine how these two fly species respond to different shades of green and yellow. Three shade levels of each colour were presented under controlled conditions. The results showed that *L. cuprina* was most attracted to medium intensity yellow, while *Ch. rufifacies* was most attracted to medium intensity green. These findings improve our understanding of how blowflies respond to colour and may be used to design more effective fly traps.

## 1. Introduction

One approach to controlling nuisance flies in the environment involves understanding their visual and olfactory sensing behaviour and how this may be utilised to attract them to traps [1]. These traps are generally designed to catch large numbers of flies. A decrease in fly populations aids in the reduction in myiasis of farm animals and subsequently diminishes the cost of this disease on human communities [2].

A review of adult flies typically displaying positive phototaxis, a tendency to move toward light stimuli, was documented by Gorostiza et al. in 2016 [3]. The strength and direction of this response depended on the physical properties of the light, such as intensity, dominant wavelength, and spectral purity, which corresponded perceptually to brightness, hue, and saturation [4]. The response was also modulated by age-dependent changes and is strictly governed by circadian rhythms [5].

Like most insects, the compound eye of a fly is a combination of many ommatidia, each containing eight photoreceptor cells (R1–R8) [6]. The diversity of these receptor cells supports an array of visual adaptations: nocturnal vs. diurnal, predator vs. prey, and landlubber vs. aerialist [6]. These photoreceptor cells are arranged in two ways within an ommatidium, as R1 to R6 in the outer region, extending the entire retina length, and R7 and R8 in the middle, on top of each other. Previous electrophysiological recordings of the *Lucilia* species showed that R1–R6 cells exhibited peak sensitivity to green wavelength. The remaining two cells, R7 and R8, are categorised into two groups—pale (p) and yellow (y)—based on their appearance in transmitted light, hence identified as R7p, R7y, R8p, and R8y, and they peaked in sensitivity for blue wavelengths [7].

Commercial fly traps utilised to catch myiasis-causing flies such as *Lucilia cuprina* Wiedemann and *Lucilia sericata* (Meigen) are constructed using different coloured surfaces, mostly with wavelengths between 495 and 590 nm, which represent the green and yellow components of the visible spectrum. Yellow traps sold commercially are Red Top Flycatcher^®^, Buzz Disposable Fly Catch^®^, Agrilure Trap^®^, Luci Trap^®^, and Green Killer Fly paper traps^®^. In contrast, green coloured commercial traps include Easy Traps^®^, Hanging-type outdoor Ranch fly traps^®^, Fly Eater fly traps^®^, and Rescue Fly Pad sticky traps ^®^ [2].

The earliest known study examining colour attraction in *L. cuprina* was conducted by Fukushi (1989) [8], who reported that this species was most attracted to yellow, followed by blue, red, and green. Another laboratory investigation by Wall and Smith (1996) [9] also explored colour preferences in *L. cuprina* and *L. sericata*, respectively, under conditions excluding olfactory cues, and similarly found that yellow was generally more attractive than green, capturing higher numbers of flies. More recently, Brodie et al. (2014 and 2015) [10,11] conducted a detailed laboratory study on gravid female Calliphoridae, examining the combined effects of visual and olfactory cues, and observed a preference for darker colours such as dark red and black.

Hue represents the qualitative aspect of colour and is primarily determined by the dominant wavelength of light reflected from a surface. The reflectance spectrum of a material describes the proportion of light reflected at each wavelength, and the wavelength with the highest reflectance largely defines the perceived hue [12]. The present study focused on females of two species, representing primary and secondary myiasis-causing flies: *L. cuprina* (a primary species) and *Ch. rufifacies* (Macquart) (a secondary species). Importantly, this study isolates the effect of visual cues independently of olfactory influences, providing an understanding of the number of flies attracted to these different reflectance levels in green and yellow hues, as well as their proximity to the light source.

## 2. Materials and Methods

### 2.1. Fly Colony

Two laboratory colonies of *L*. *cuprina* and *Ch*. *rufifacies* were established for the experiments. The colonies were initiated from a mixed assemblage of adult flies collected from a rural sheep farm in Irishtown (−31.5896172 latitude and 116.6059032 longitude), Western Australia, using hanging-type ranched fly traps^®^ (18 (L) × 28 (W) × 45 (H) cm). Experimental flies were obtained from the second to fourth laboratory generations to maintain genetic consistency while minimising potential laboratory adaptation effects. All fly colonies were housed in an insectary set at 24 °C, 12:12 L:D and 60% R.H. The field caught flies were placed in cages (BugDorm^®^—30 × 30 × 30 cm, Taichung, Taiwan), each housing 60 to 70 flies, and were provided with a diet consisting of sugar and milk (100 mL), along with 100 g of sheep meat and 20 mL calf blood soaked into tissue paper, serving as an egg-laying substrate. The following day, the eggs were isolated and allowed to hatch. Once the larvae reached the third instar stage, the two species were identified [13] and placed in separately labelled insect cages. A total of eight colony cages were set up. Two cages (one for each species) were used to place flies following each experiment and six cages (three for each species) were used to provide the flies required in each trial. All aspects of colony maintenance followed the methods outlined in Bambaradeniya et al. (2023) [14].

### 2.2. Setup for Choice Test

**Figure 1:**
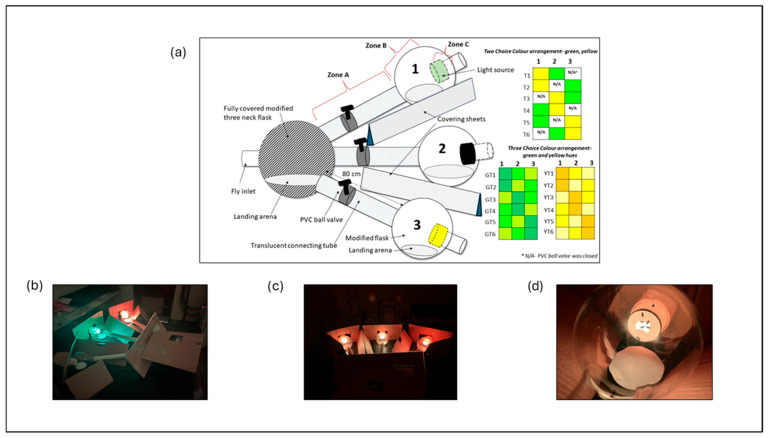
Experimental setup used for fly colour-attraction assays. (**a**) Schematic diagram showing central release chamber was connected to three landing arenas. Light sources illuminated colour treatments (green and yellow reflectance levels) for one, two and three-choice tests; (**b**) angled view showing illuminated green and yellow treatments (before being fully covered); (**c**) frontal view of the three-choice arrangement; and (**d**) top-down view of a landing arena with flies.

A glass apparatus was constructed for the choice test by connecting a 2 L three-necked flask (main flask), with its base removed, to three 1 L subsidiary flasks, also with their bases removed. Each subsidiary flask was attached to the main flask, using an 80 cm long translucent PVC tube (Figure 1). All PVC tubes featured a 25 mm PVC ball valve (tap) situated 20 cm away from the three openings of the three-neck flask. Circular cardboard pads were positioned at the bottom of each subsidiary flask to facilitate fly perching. Three Wi-Fi-enabled smart bulbs (Mirabella Genio GLS Wi-Fi ES 9 W, 800 lumens, 2700–6500 K, Mirabella International Pty Ltd., Dandenong South, VIC, Australia) were attached to the open bases of the subsidiary flasks, using three paper cups. Each cup had a square opening (1.5 × 1.5 cm) cut into the bottom, allowing only a specific light to illuminate the interior of each flask.

To prevent incoming light, triangular-shaped cardboard sheets were inserted between each flask, and the entire three-neck flask was wrapped in a black-coloured polythene sheet. Hence, the flies placed inside the main flask can see light only through the openings of the three necks into each flask. This glass apparatus was assembled horizontally on a table covered with a black tablecloth, and placed in a dark room with a temperature maintained at 27 °C. After every three trials, this setup was deconstructed and washed with distilled water and air-dried prior to being reassembled.

### 2.3. Fly Selection for Each Experiment

All experiments were conducted using 13- to 20-day-old female flies from each species. A total of 20 flies were chosen at random for each experiment involving different colour reflectance treatments. Female flies were determined by observing the intraocular distance and then captured and transferred to separate insect cages [15]. This process took 10 min to complete. After collecting 20 females, they were exposed to white light (4000 K bulb) and left without food for a one-hour period prior to introducing them into the glass apparatus. Notably, each group of 20 flies was used for one experiment, then returned to a colony cage, and played no further part in the experiment. Between each trial, flies were placed in the main flask followed by a standardised 20 min acclimation period under completely dark conditions. This was to ensure the behavioural consistency of flies at the starting point of each trial. After conducting each trial, flies were returned to the separate colony and were not reused.

### 2.4. Colour Reflectance Level Selection

Colour reflectance treatments were selected and standardised following the principles outlined by Jha (2010) [12]. Six reflectance levels were chosen to represent two major hue categories commonly used in insect behavioural studies: green (≈495–560 nm) and yellow (≈565–590 nm). Within each hue, these selected three reflectance levels were defined as light, moderate, and dark.

The colours were generated using a broad-spectrum Wi-Fi-controlled LED light source (Mirabella Genio GLS Wi-Fi ES 9 W, 800 lumens, 2700–6500 K). The hue, saturation, and brightness were adjusted using the Mirabella Genio mobile application, keeping saturation and brightness constant at 50% across all treatments to ensure uniform luminance conditions.

The selected colour samples were then matched with corresponding Munsell notations using the Munsell Book of Color (Munsell Color Science Laboratory, Rochester Institute of Technology, Rochester, NY, USA) [16]. Approximate reflectance intensities (as the percentage of reflected light at dominant wavelengths) were determined using ColorMine^®^ software (v. 1.1.3, http://colormine.org/) [17], which converts Munsell values into spectral reflectance data (%). These values provided a quantitative basis for classifying each hue into light, moderate, and dark reflectance levels.

### 2.5. Fly Attraction Number Count

After the flies had settled in the main flask, the appropriate valves were opened according to the designated treatment. The number of flies present in each zone (A, B, and C) was recorded at 5, 15, and 30 min intervals. The total fly number was defined as the number of flies recorded after 30 min, representing the sum of individuals present across all three zones under each reflectance intensity treatment.

### 2.6. Reflectance-Based Choice Tests

Three behavioural experiments were designed to evaluate fly attraction responses under varying light reflectance levels. These included one-way, two-way, and three-way choice tests, each differing in the number of available reflectance level options and treatment combinations. The one-way choice test served as an intermediate reference, providing a behavioural baseline for comparison with the more complex two- and three-choice experiments (Table 1).

#### 2.6.1. One-Way Choice Experiment (Experiment 1)

The one-way choice experiment was conducted to evaluate the direct visual response of female flies to moderate green (5G 6/8) and moderate yellow (5Y 8/8) light reflectance levels. The glass apparatus (Figure 1) was configured so that only the middle subsidiary flask was illuminated during each trial, and the corresponding ball valve was selectively opened to expose flies to a single light source. Three replicates were performed for each species, with twenty female flies per replicate, resulting in a total of 120 flies tested in both green and yellow (Table 1).

#### 2.6.2. Two-Way Choice Experiment (Experiment 2)

The two-way choice experiment followed the same setup and procedure as Experiment 1. In this test, flies were simultaneously offered a choice between two illuminated subsidiary flasks, using combinations of moderate green (5G 6/8) and moderate yellow (5Y 8/8) reflectance. During each trial, one PVC ball valve remained closed to restrict access to a single flask, allowing flies to choose between the two available light sources.

The position of each light treatment was alternated between trials to minimise positional bias. Six distinct treatment configurations were tested (T1–T6; Figure 1), with each treatment replicated three times for both species, resulting in a total of 36 replicates (18 per species) (Table 1).

#### 2.6.3. Three-Way Choice Experiment (Experiment 3)

The three-way choice experiment followed the same general procedure as Experiment 1 but was extended to include three distinct reflectance intensities for both colour treatments. The green reflectance levels tested were light (5GY 8/10), moderate (5G 6/8), and dark (5G 4/6) green, while for yellow, they were light (5Y 9/10), moderate (5Y 8/8), and dark (10YR 7/8) yellow. During each trial, all three subsidiary flasks were illuminated simultaneously, allowing flies to choose between the three light treatments. Six treatment configurations were tested for each colour, with three replicates conducted per species, resulting in 72 total replicates (36 for each species) (Table 1).

### 2.7. Data Analysis

All statistical analyses were performed in R (version 4.5.1). The normality and homogeneity of variance were checked prior to testing. Differences in movement frequencies under one-way choice conditions (Experiment 1) were assessed using chi-square tests of independence. For attraction assays (Experiments 2 and 3), differences in the number of individuals attracted to different reflectance intensities were evaluated using paired *t*-tests or one-way ANOVA, as appropriate. Where significant effects were detected, post hoc Tukey’s HSD tests were applied to identify pairwise differences. Effect sizes (Cohen’s *d*) were calculated for *t*-tests to quantify the magnitude of differences. Descriptive statistics (mean ± SD) were used to summarise the data, and all figures were generated in R using the ggplot2 package (version 4.5.1).

## 3. Results

### 3.1. Determination of Reflectance Levels in Green and Yellow Hues

**Figure 2:**
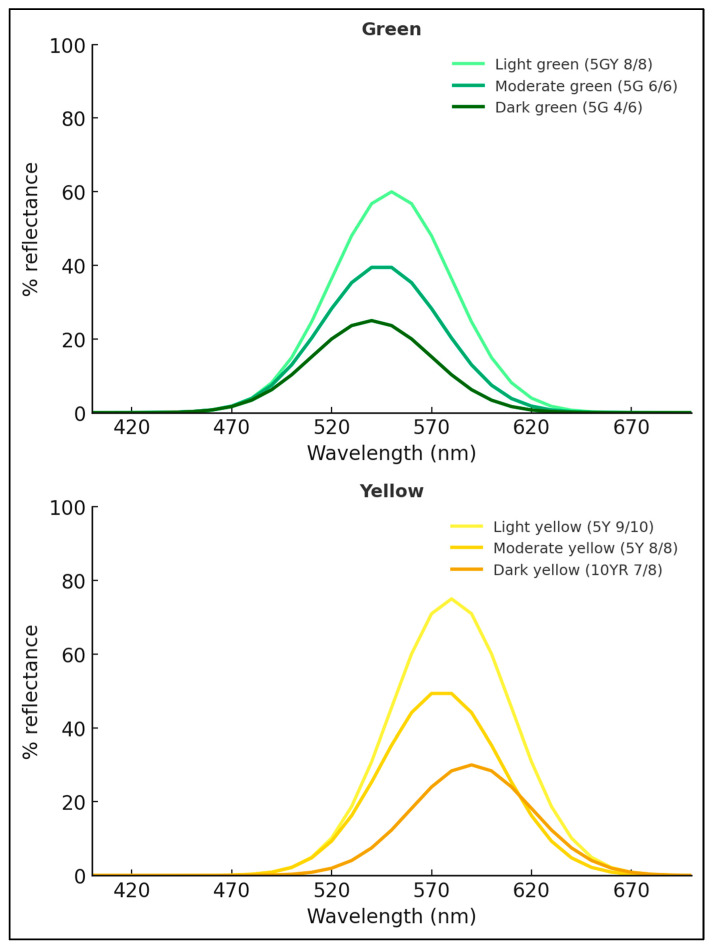
Reflectance of green and yellow hues, showing light, moderate, and dark levels across 400–700 nm.

The reflectance levels of the selected green and yellow hues were defined using three complementary methods: (1) reference colours identified within the Mirabella Genio mobile application, (2) corresponding Munsell notations, and (3) quantified reflectance values (%) with associated approximate dominant wavelengths (Table 2). The relationships among these parameters were further illustrated graphically, showing the variation in reflectance (%) across the visible wavelength range (400–700 nm) for each hue (Figure 2).

### 3.2. Experiment 1

**Figure 3:**
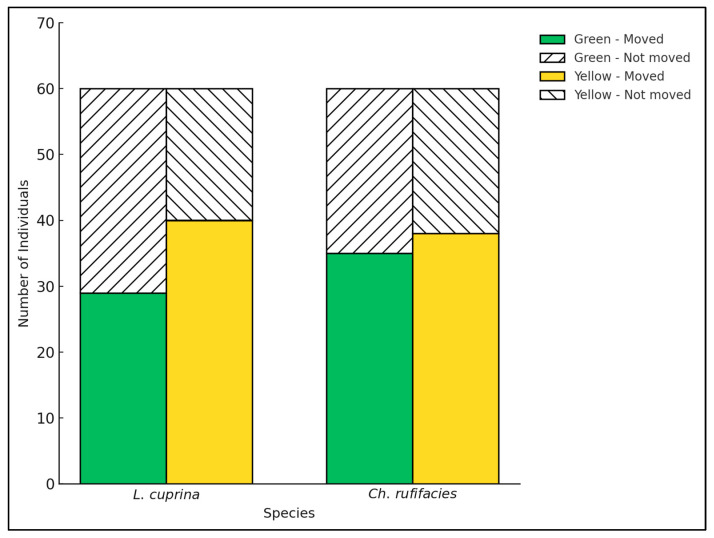
Movement response of *L. cuprina* and *Ch. rufifacies* females under one-way choice conditions toward two reflectance colours: moderate green (5G 6/8) and moderate yellow (5Y 8/8).

A chi-square test of independence was conducted to assess whether moderate green and yellow influenced the movement behaviour of *L. cuprina* and *Ch. rufifacies* in a one-way choice trial. *Lucilia cuprina* yielded no statistically significant association between reflectance and movement (χ^2^ = 3.27, *p* = 0.070). The mean number of individuals that moved toward the moderate yellow surface was slightly higher (mean = 40) than toward the moderate green (mean = 29), suggesting a weak attraction (Figure 3). Similarly, for *Ch. rufifacies*, movement toward the two reflectance colours, moderate green (mean = 35) and yellow (mean = 38) did not differ significantly (χ^2^ = 0.11, *p* = 0.74), indicating comparable attraction to both reflectance intensities (Figure 3). Overall, while both species were active during the observation period, the colour did not significantly influence movement.

### 3.3. Experiment 2

At the 30 min observation period, a paired *t*-test was conducted to compare the number of individuals attracted to the two reflectance colours of either moderate yellow or moderate green for each species. *L. cuprina* showed no significant difference between the two reflectance intensities (*t* = 1.61, *p* = 0.25), indicating that both colours elicited a similar level of attraction. In contrast, *Ch. rufifacies* exhibited a statistically significant preference for the green reflectance (*t* = −15.23, *p* = 0.004).

**Figure 4:**
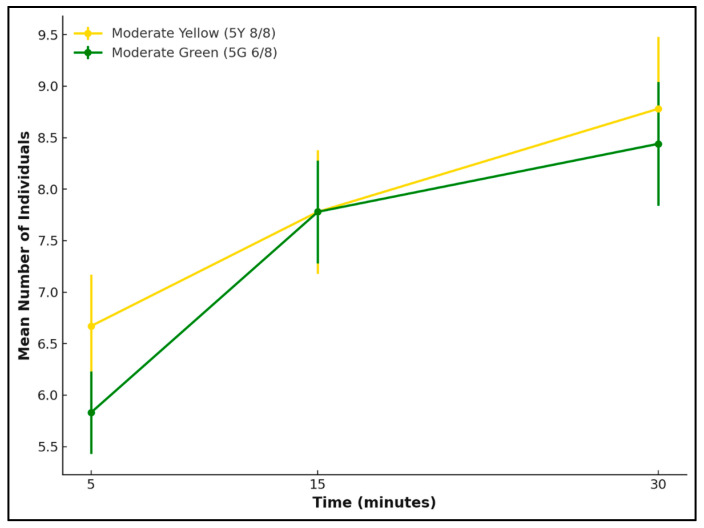
Attraction of *L. cuprina* females to two reflectance colours—moderate yellow (5Y 8/8) and moderate green (5G 6/8)—over a 30 min period. Lines represent mean ± SD values at 5, 15, and 30 min.

**Figure 5:**
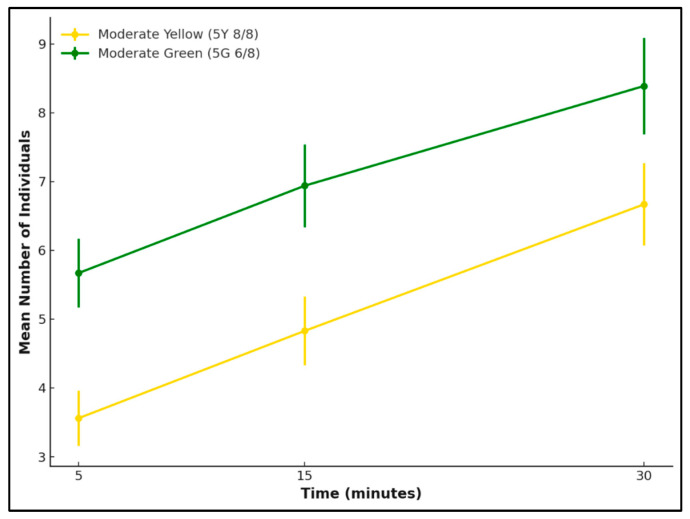
Attraction of *Ch. rufifacies* females to two reflectance colours—moderate green (5G 6/8) and moderate yellow (5Y 8/8)—over a 30 min period. Lines represent the mean number of individuals (±SD) recorded at 5, 15, and 30 min.

Over the 30 min observation period, both *L. cuprina* and *Ch. rufifacies* showed an overall increase in attraction to the tested reflectance, but the temporal patterns and reflectance selection differed between the two species (Figure 4 and Figure 5). For *L. cuprina*, the attraction increased gradually for both the moderate yellow and moderate green, with mean numbers of individuals rising within 5 min compared with 30 min. Although *L. cuprina* consistently showed slightly higher counts on the yellow surface, the difference between reflectance remained minor throughout the observation period (Figure 4). In contrast, *Ch. rufifacies* exhibited a stronger and more consistent response to the moderate green, with the mean number of individuals increasing sharply over time (Figure 5). Overall, the results indicate species-specific visual attraction, with *L. cuprina* responding similarly to both yellow and green reflectance, while *Ch. rufifacies* showed a clear tendency for attraction to green reflectance as time progressed.

#### Zonal Attraction

The settlement patterns for *L. cuprina* differed notably among zones within each reflectance treatment. Under moderate green reflectance, the mean number of flies increased sharply from Zone A (0.50 ± 0.71) and Zone B (0.78 ± 0.88) to Zone C (7.06 ± 3.10). Similarly, under moderate yellow reflectance, Zone C (8.11 ± 2.63) attracted substantially more flies than Zone A (0.50 ± 0.99) and Zone B (0.17 ± 0.51) (Table 2). Independent *t*-tests comparing zones within each reflectance showed that Zone C attracted significantly more flies than both Zones A and B under green (*t* = 5.27, *p* = 0.006, *d* = 2.64) and yellow (*t* = 6.02, *p* = 0.004, *d* = 3.02) colours. However, corresponding zone comparisons between green and yellow reflectance treatments (e.g., Green C vs. Yellow C) revealed no significant difference (*t* = −0.07, *p* = 0.95, *d* = −0.04).

A similar though less pronounced trend was observed for *Ch. rufifacies*. Mean fly numbers increased from Zone A (1.78 ± 1.26) and Zone B (2.50 ± 1.47) to Zone C (3.94 ± 2.31) for green reflectance. Means also rose toward the terminal zone—Zone A (1.67 ± 1.57), Zone B (1.61 ± 1.46), and Zone C (3.39 ± 1.54) (Table 3)—for yellow reflectance. Independent *t*-tests confirmed significantly higher numbers in Zone C compared with Zones A and B for both reflectance treatments (*t* = 3.16, *p* = 0.034, *d* = 1.58 for green; *t* = 2.92, *p* = 0.043, *d* = 1.46 for yellow). Reflectance comparisons for corresponding zones indicated no significant difference (*t* = 0.39, *p* = 0.72, *d* = 0.18).

Overall, both *L. cuprina* and *Ch. rufifacies* exhibited a tendency to settle near the terminal (C) zone, nearest to the light source, regardless of reflectance.

### 3.4. Experiment 3

#### 3.4.1. Green

A one-way ANOVA was used to assess whether attraction differed significantly between the three green reflectance levels: light, moderate, and dark for *L. cuprina* and *Ch. rufifacies* after 30 min. No significant differences were observed for either species (*L. cuprina*: *F* = 1.21, *p* = 0.32; *Ch. rufifacies*: *F* = 0.24, *p* = 0.79).

Attraction was relatively uniform across reflectance levels for *L. cuprina*, with a slight tendency toward the moderate green surface, which consistently recorded the highest mean captures at 5, 15, and 30 min. The dark green also elicited moderate attraction, while the light green was least preferred. Although the differences were not statistically significant, the pattern suggests that *L. cuprina* responds broadly to green hues, with only a mild preference for mid-range reflectance intensities.

Attraction increased progressively across time in all green reflectance treatments for *Ch. rufifacies*, but was greatest when presented with moderate green, followed by dark and light green. This clearer preference indicates that intermediate reflectance levels may provide optimal brightness and contrast cues for orientation in this species.

**Figure 6:**
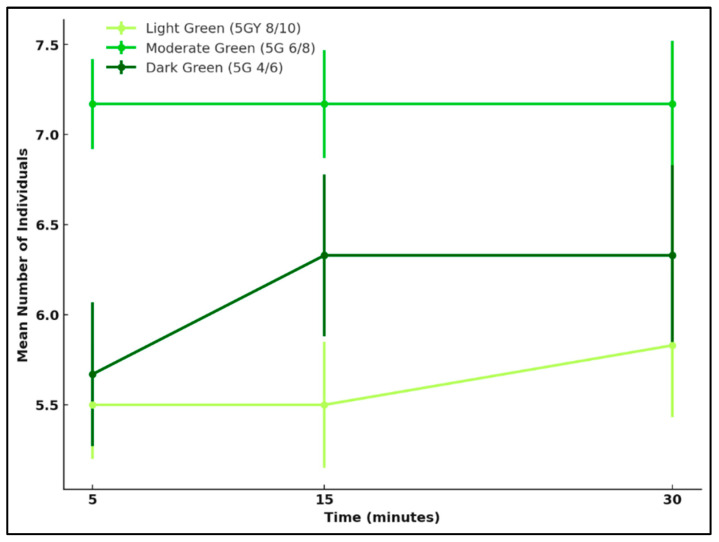
Attraction of *L. cuprina* adults to three green reflectance intensities—light green (5GY 8/10), moderate green (5G 6/8), and dark green (5G 4/6)—over a 30 min period. Lines represent the mean number of individuals (±SE) recorded at 5, 15, and 30 min.

**Figure 7:**
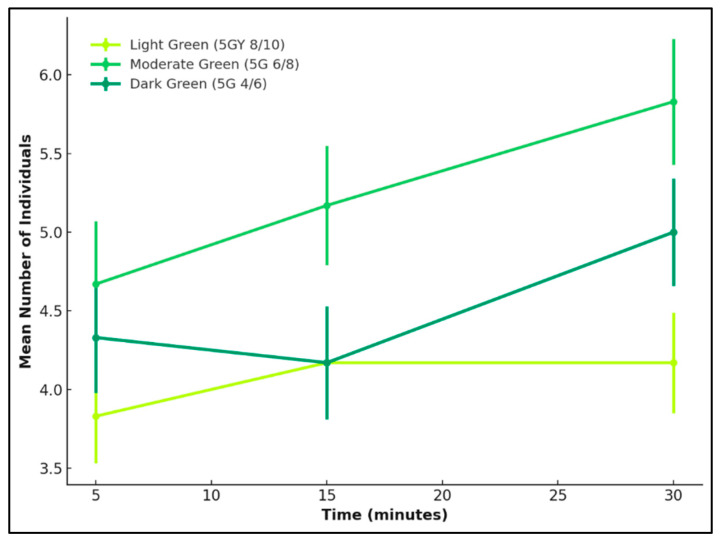
Attraction of *Ch. rufifacies* females to three green reflectance intensities—light green (5GY 8/10), moderate green (5G 6/8), and dark green (5G 4/6)—over a 30 min period. Lines represent mean numbers of individuals (±SE) recorded at 5, 15, and 30 min.

Overall, both species were sensitive to variation in green reflectance, showing the greatest attraction to the moderate green colour. However, *Ch. rufifacies* displayed a more distinct and selective preference for this mid-range colour, whereas *L. cuprina* responded more evenly across the three green levels, with only a small bias toward the moderate green colour (Figure 6 and Figure 7).

#### 3.4.2. Yellow

A one-way ANOVA was conducted to assess whether attraction differed significantly among the three yellow reflectance levels: light, moderate, and dark yellow. No significant differences were detected for either species (*L. cuprina*: *F* = 1.92, *p* = 0.18; *Ch. rufifacies*: *F* = 0.15, *p* = 0.86).

Attraction remained relatively consistent throughout the 30 min observation period for *L. cuprina*. Although their attraction was slightly higher to light yellow, the differences were small and not statistically significant, suggesting a uniform response across the yellow reflectance levels.

Attraction was again highest to moderate yellow for *Ch. rufifacies*, showing a subtle tendency toward intermediate reflectance. However, differences were not significant, indicating that reflectance intensity had little or no influence on attraction.

**Figure 8:**
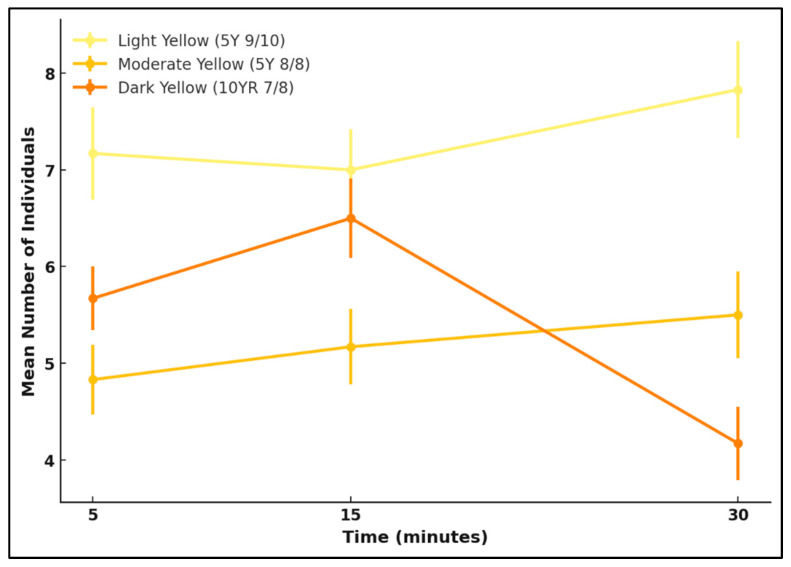
Attraction of *L. cuprina* adults to three yellow reflectance intensities—light yellow (5Y 9/10), moderate yellow (5Y 8/8), and dark yellow (10YR 7/8)—over a 30 min period. Lines represent the mean number of individuals (±SE) recorded at 5, 15, and 30 min.

**Figure 9:**
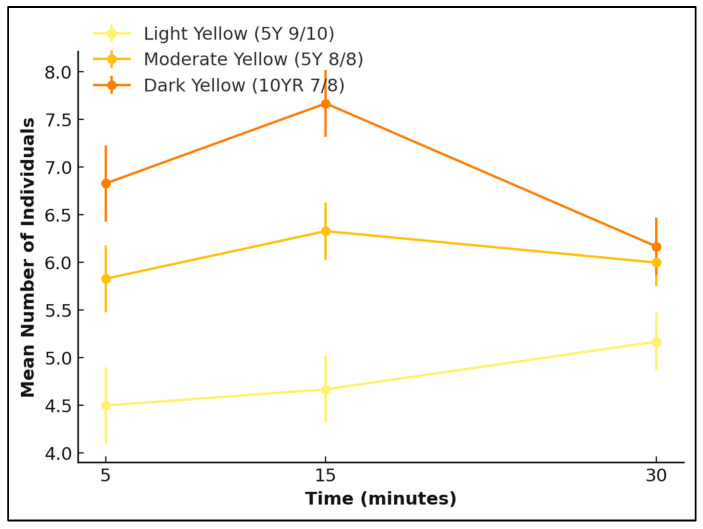
Attraction of *Ch. rufifacies* adults to three yellow reflectance intensities—light yellow (5Y 9/10), moderate yellow (5Y 8/8), and dark yellow (10YR 7/8)—over a 30 min period. Lines represent the mean number of individuals (±SE) recorded at 5, 15, and 30 min.

Overall, both species responded similarly across yellow reflectance intensities, with *L. cuprina* showing a stable pattern and *Ch. rufifacies* demonstrating a slightly stronger preference for moderate yellow colours (Figure 8 and Figure 9).

#### 3.4.3. Zonal Attraction in Green and Yellow

After 30 min, both *Lucilia cuprina* and *Chrysomya rufifacies* displayed distinct settling patterns across zones in green and yellow reflectance treatments.

Adults consistently concentrated in Zone C for *L. cuprina*, showing strong zone settling behaviour in both reflectance categories. Mean numbers in Zone C ranged from 5.17 ± 2.55 to 6.56 ± 3.31, whereas Zones A and B attracted very few individuals (≤0.61 ± 1.04). A one-way ANOVA confirmed significant differences among zones for both green (*F* = 43.69, *p* < 0.001) and yellow reflectance (*F* = 23.59, *p* < 0.001). These results indicate that *L. cuprina* exhibited a pronounced preference for the terminal Zone C, regardless of the reflectance level, suggesting a strong orientation response toward light.

The overall attraction was lower but followed a similar spatial pattern for *Ch. Rufifacies.* There were no significant differences detected between zones (F = 0.49, *p* = 0.62) when subjected to green reflectance (Zones A (1.28 ± 0.96), B (1.22 ± 1.11), and C (2.50 ± 1.98)). In contrast, when subjected to yellow reflectance, significant differences occurred among zones (*F* = 44.40, *p* < 0.001), with markedly higher numbers in Zone C (4.44 ± 2.55) than in Zones A (0.68 ± 0.91) and B (0.48 ± 0.84) (Table 4).

*Lucilia cuprina* aggregated in the terminal zone under both green and yellow reflectance, while *Ch. rufifacies* showed a weaker response to green but a clear increase in attraction toward Zone C under yellow reflectance. These findings suggest that both species are influenced more by spatial position relative to the light source than by differences in reflectance intensity.

## 4. Discussion

Across the three experiments, both *Lucilia cuprina* and *Chrysomya rufifacies* exhibited attraction patterns influenced by reflectance intensity and spatial position, relative to the light source. Although neither species showed strong colour discrimination, *L. cuprina* demonstrated a trend toward the moderate yellow reflectance, while *Ch. rufifacies* displayed a statistically significant preference for the moderate green reflectance. This was demonstrated in Experiment 1, where movement responses revealed no significant association between colour and attraction for either species, though *L. cuprina* showed an inclination toward yellow. Experiment 2 confirmed this trend, with *L. cuprina* showing a higher attraction to yellow, whereas *Ch. rufifacies* was attracted to green over time. Experiment 3 further demonstrated that both species responded most to moderate reflectance intensities, with minimal attraction to light or dark reflectance. These findings align with Fukushi (1989) [8], who reported that *L. cuprina* was most attracted to yellow, supporting the present observation of yellow preference. However, this conflicts with Lunau’s (2014) [6] study on the electrophysiological recordings of *Lucilia*, whereby six of the R cells of the ommatidia had peak sensitivity to green wavelengths. The remaining two cells were sensitive to pale and yellow, which may well be dominant in transmitted light, as suggested in [6]. Unfortunately, no comparable data for *Ch. rufifacies* is documented in the literature, making this study among the first to document its distinct preference for green reflectance under controlled laboratory conditions.

Notably, zone-based analyses revealed that most individuals of both species accumulated in the terminal zone nearest to the light source (Zone C), irrespective of reflectance, indicating that spatial orientation cues exerted a stronger influence on settling behaviour than hue alone. These insights provide valuable directions for future research, particularly in exploring how fly attraction or avoidance behaviours are modulated by additional colour-related parameters such as brightness, saturation, and intensity in relation to spatial orientation thresholds. Understanding these interactions could be utilised in the development of light and colour-based management strategies to deter fly aggregation and reduce their presence in livestock and waste environments.

Compared with previous studies, several methodological refinements were implemented in the present investigation. Earlier colour preference studies typically controlled for odour by standardising a common olfactory source to isolate visual effects [10]. In contrast, this study was conducted in a fully enclosed, odour-free room without any specific odour-generating source. The experimental glass setup had only an opening covered by a single mesh allowing minimal airflow, ensuring that the observed responses were not influenced by external scent cues. Additionally, unlike previous experiments that used uniform ambient illumination such as fluorescent ceiling lights to maintain equal reflectance across treatments [10,18], this study employed only the tested coloured light sources, with each fully enclosed to prevent spectral overlap. To minimise flicker-related artefacts, zero-flicker Wi-Fi smart bulbs were used to provide stable illumination, as flies possess high temporal visual sensitivity [19]. Furthermore, while earlier studies [8,9] quantified colour reflectance using spectrophotometric measurements, the present study employed computer-based reflectance analysis, providing a flexible approach for determining the intensity and consistency of light cues presented to the flies.

Most insect colour or light preference studies include a white or neutral background as a control to measure attraction responses [10]. However, this approach is not universally applied; for instance, Fukushi (1990) [20] examined the attraction of *L. cuprina* to three shades of grey without incorporating white as a control. The present study was specifically designed to compare relative responses among reflectance levels within the green and yellow spectra, rather than to determine attraction to colour per se. Accordingly, moderate green and moderate yellow were selected as internal reference colours instead of a colourless control. To minimise potential bias associated with non-neutral references, flies were tested in all possible colour combinations between moderate green and moderate yellow, using a two-choice design. This ensured that each colour served both as a treatment and as a comparison, providing a balanced and internally consistent measure of relative attraction. In practical field trapping systems, flies are more likely to encounter coloured surfaces such as green or yellow panels rather than neutral or white backgrounds; therefore, employing internal references that represent commonly used trap colours provides a realistic and ecologically relevant comparison. While including a colourless control could have enabled assessment of absolute attraction to colour, the present approach offers valuable insight into hue dependent visual responses under controlled field-relevant conditions. Future research could build upon this framework by incorporating a neutral baseline to further clarify the role of colour contrast in fly attraction.

The main objective of this study was to record the response of the two species to different colour intensities of the two most used fly trap colours. It is important to note, however, that these traps do not function solely as colour or light attractants, as they are always combined with olfactory stimuli, such as a piece of rotten meat or volatile organic compounds [2]. Conducting a future study that combines these factors with colour and light could provide a more precise understanding of colour preference. In such studies, it would be essential to keep these other factors constant while varying the colour intensities.

## Figures and Tables

**Table 1:** Summary of experimental designs for one-, two-, and three-way choice experiments assessing fly attraction to the light reflectance levels. Experiments varied in the number of illuminated flasks, treatment combinations, and replicates per species.

Experiment	Choice Test	Reflectance Intensities	No. of Subsidiary Flasks	Light Source Configuration	Replicates/Species	No. of Treatments/Species	Total Replicates/Species
1	One-way choice	Moderate green (5G 6/8) and moderate yellow (5Y 8/8)	1 (middle flask only)	Single flask illuminated/trial	3	1	3
2	Two-way choice	Moderate green (5G 6/8) and moderate yellow (5Y 8/8)	2 (one closed per trial)	Two flasks illuminated simultaneously	3	6	18
3	Three-way choice	Green: light (5GY 8/10), moderate (5G 6/8), dark (5G 4/6) Yellow: light (5Y 9/10), moderate (5Y 8/8), dark (10YR 7/8)	3	All 3 flasks illuminated simultaneously	3	6	36

**Table 2:** Description of colour treatments used in the experiment, showing Munsell notation and corresponding reflectance intensity ranges for each hue. Reflectance values represent approximate percentages of light reflected at dominant wavelength bands, as determined using ColorMine^®^ software (http://colormine.org).

Colour	Colour Reference	Munsell Notation	Reflectance Intensity	Dominant λ (nm, Approx.)
Light green	* 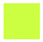 *	≈5GY 8/10	≈45–60%	550
Moderate green	* 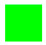 *	≈5G 7/10	≈25–40%	545
Dark green	* 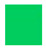 *	≈5G 4/6	≈15–25%	540
Light yellow	* 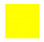 *	≈5Y 9/10	≈60–75%	580
Moderate yellow	* 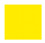 *	≈5Y 8/8	≈35–50%	575
Dark yellow	* 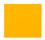 *	≈10YR 7/8	≈15–30%	590

**Table 3:** Mean (±SD, n = 3) number of *L. cuprina* and *Ch. rufifacies* flies settled in each zone (A–C) under moderate green and yellow reflectance after 30 min of exposure (Experiment 2). Superscript letters indicate significant differences among zones within the same reflectance treatment, based on independent-sample *t*-tests (*p* < 0.05).

Species	Colour	Zone		
		*A*	*B*	*C*
*L. cuprina*	Green	0.50 ± 0.71 ^a^	0.78 ± 0.88 ^a^	7.06 ± 3.10 ^b^
	Yellow	0.50 ± 0.99 ^a^	0.17 ± 0.51 ^a^	8.11 ± 2.63 ^b^
*Ch. Rufifacies*	Green	1.78 ± 1.26 ^a^	2.50 ± 1.47 ^a^	3.94 ± 2.31 ^b^
	Yellow	1.67 ± 1.57 ^a^	1.61 ± 1.46 ^a^	3.39 ± 1.54 ^b^

**Table 4:** Mean (±SD, n = 3) number of *L. cuprina* and *Ch. rufifacies* adults recorded in Zones A–C under different green and yellow reflectance intensities after 30 min of exposure. Different superscript letters within a row indicate significant differences among zones, based on one-way ANOVA with post hoc comparison (*p* < 0.05).

Species	Colour Hue	Zone		
		*A*	*B*	*C*
*L. cuprina*	Light Green	0.06 ± 0.24 ^a^	0.44 ± 0.70 ^a^	5.94 ± 2.82 ^b^
	Moderate Green	0.00 ± 0.00 ^a^	0.61 ± 1.04 ^a^	5.50 ± 2.48 ^b^
	Dark Green	0.06 ± 0.17 ^a^	0.61 ± 1.04 ^a^	6.33 ± 2. 43 ^b^
	Light Yellow	0.44 ± 0.62 ^a^	0.56 ± 0.86 ^a^	6.56 ± 3.31 ^b^
	Moderate Yellow	0.11 ± 0.32 ^a^	0.39 ± 0.78 ^a^	5.17 ± 2.55 ^b^
	Dark Yellow	0.17 ± 0.38 ^a^	0.61 ± 0.85 ^a^	5.22 ± 2.62 ^b^
*Ch. rufifacies*	Light Green	1.28 ± 0.96 ^a^	1.22 ± 1.11 ^a^	2.50 ± 1.98 ^b^
	Moderate Green	1.78 ± 1.52 ^a^	0.72 ± 0.96 ^a^	2.89 ± 2.30 ^b^
	Dark Green	0.78 ± 1.06 ^a^	1.11 ± 1.28 ^a^	2.44 ± 1.98 ^b^
	Light Yellow	0.56 ± 1.04 ^a^	0.78 ± 0.81 ^a^	4.44 ± 2.55 ^b^
	Moderate Yellow	0.89 ± 0.83 ^a^	1.00 ± 0.90 ^a^	3.94 ± 2.24 ^b^
	Dark Yellow	0.61 ± 0.85 ^a^	0.78 ± 0.81 ^a^	4.00 ± 2.93 ^b^

## Data Availability

The original contributions presented in this study are included in the article. Further inquiries can be directed to the corresponding author.
